# Loot

**Published:** 1871-05

**Authors:** 


### Loot.

***The Treatment of Internal Strangulation of the Bowel (Ileus Volvulus).***

M. Tillaux, of the Hôpital St. Antoine, Paris, recognizes two forms of internal strangulation, the true and the false: the former arising from a physical cause, the latter from a dynamic cause; or, in other words, the former being produced by a mechanical obstacle to the passage of the contents of the intestine, the latter by a contraction or a paralysis of the coats of the intestine themselves. In the present essay he speaks only of the false variety, and shows that one of the most frequent causes of this state is acute peritonitis, either of a primary nature or consecutive upon a perforation of the intestine. Is it possible, he asks, to distinguish between these last two? He gives a series of cases where operations have been undertaken by distinguished surgeons, in which the peritoneal cavity was opened and yet no strangulation discovered, peritonitis alone being present; whilst in other cases internal strangulation has been judged to be present when the real disease was a calculus impacted in the biliary duct. Both in primary peritonitis and in that resulting from perforation the pain occurs suddenly, but its characters are not identical in the two cases. In strangulation the pain is spontaneous and but little increased by palpation or pressure of the abdomen, whilst in peritonitis it is excessive. Vomiting occurs at the outset in both cases; but in peritonitis it rarely becomes fæcal; in strangulation it may be bilious at first, but rapidly becomes fæcal. Constipation is obstinate and complete in internal strangulation, but in acute peritonitis some evacuations usually occur in response to the purgatives that are often given originally. The pulse is frequent and very small in peritonitis, even from the beginning; but these characters are prominent only in the latter stages of strangulation. The expression of the face is anxious and materially altered at an early stage of peritonitis; but in strangulation the expression of the face, though altered, yet presents a character that is difficult to express in words and is best caught at the bedside of the patient. The belly is tympanitic in both cases, but chiefly in peritonitis. When a true and complete internal strangulation occurs, the surgeon has two indications to fulfill: to remove the constriction and to give a new point of discharge for the fæces. The first is accomplished by the operation of gastrotomy, the arguments for the performance of which are materially supported by the modern practice of ovariotomy, and M. Besnier, who has paid much attention to the operation, is strongly in favor of it. M. Tillaux, however, objects to it, on the grounds, first, of the extreme difficulty of discovering the seat of constriction, and, secondly, of the inflamed condition of the intestines. The second indication is fulfilled by the operation of enterotomy, as suggested by Nelaton, who, resting on the fact that it is the small intestine that usually undergoes strangulation, makes a small opening in the right iliac region, seizes, draws forth, and opens the first loop of intestines that presents itself, and thus gives issue to the fæcal matter. M. Tillaux maintains the superiority either of this plan or that of M. Lavgier, which consists in stabbing the intestine with a trochar, as the best and most appropriate treatment. (*Bulletin général de Therapeutique*, liv. vii. 1870.)—*Practitioner.*

**Large Doses of Chloral.**

Dr. James Rodman states (*The American Practitioner*, Oct. 1870) that a nurse gave one of his patients, who was under treatment for insanity, in order to induce sleep, 270 grains of hydrate of chloral, which produced sleep for eighteen hours, without any alarming attendant symptoms.

A case is reported (*Medical Times*, Oct. 15, 1870) of a female nurse in the Philadelphia Hospital, who took 460 grains of chloral. It produced deep sleep, her respirations were 35—heavy and stertorous; pulse 140: face flushed; extremities cold and livid. The symptoms resembling those produced by an overdose of opium, she was supposed to have taken that drug, and was treated in accordance with that supposition. She entirely recovered, when she stated she had taken some medicine from a vial for the relief of headache, and it was ascertained that the medicine was a solution of chloral.

It is stated in the *Lancet* (Nov. 26, 1870), that as much as 160 grains of the hydrate of chloral were lately given by mistake in one dose to a middle-aged man in one of the London hospitals. Some alarm was felt, but no disagreeable symptoms occurred; the man slept well, and no unpleasant effects resulted.—*Medical News.*

**Oil of Peppermint as a Local Anæsthetic.**

Dr. A. Wright writes to the editor of the *Lancet* (Nov. 19, 1870), that "a few years ago I became acquainted with the fact of the natives [Chinese], when suffering with a facial neuralgia, using oil of peppermint, which they lightly apply to the seat of pain with a camel-hair pencil. Since then, in my own practice, I frequently employ oil of peppermint, in the same way, as a local anæsthetic, not only in neuralgia, but also in gout, with remarkably good results; indeed, the relief from pain I have found to be almost instantaneous."

It is worthy of note that some Chinese pharmaceutists in San Francisco and New York have been selling a remedy for neuralgia, which has gained some repute. It is a liquid put up in very small vials, holding about half a drachm each, which are sold at an exorbitant price. The liquid has a strong smell of peppermint, and is in all probability the oil of that plant.—*Medical News.*

Twenty-five years ago, at least, the oil of peppermint was a common application in neuralgia. We recollect being informed by Dr. Stewart, one of the oldest practitioners of Michigan, now living, that he learned its use from the noted Dr. White, of Cherry Valley, N. Y., many of whose students, now venerable with years, have always been in the habit of using it for this purpose. The pity is that it is not more successful, yet we scarcely take up a secular paper but has this item as something new from the "heathen Chinee." As a new thing it will do to put alongside of the St. Louis "substitute for the stomach pump," which our contemporaries are still publishing as a novelty, though we last year published its origin dating nearly fifty years ago. How long will it take, at this rate, to get back to the medical philosophy and appliances of five hundred years ago, which are now the prevailing Celestial style?

***Enlargement of the Uterus.***

Dr. Atthill read a paper on this disease, at a late meeting of the Dublin Obstetrical Society:

After passing an encomium on the uterine sound, which he considers to be not only one of the most useful, but also the safest instrument we possess, if carefully and skillfully used, he proceeded to say that enlargement of the uterus was met with in a very large percentage of those cases in which the symptoms are referable to the organs of generation in the female, a condition easily explicable when the great change which takes place in the uterus under the influence of pregnancy, and even, in a degree, at each menstrual period, is borne in mind. The following, apart from the actual existence of pregnancy, are the causes to which most frequently enlargement of the uterus is due, namely:

1. Sub-involution of the uterus after pregnancy or abortion.
2. Congestion of the uterus from sudden suppression or retardation of menstruation.
3. Acute inflammation of the uterus, or its peritoneal covering.
4. Chronic inflammation of the uterus.
5. Hypertrophy of the uterus.
6. The stimulus given to the uterus by the presence in its walls of fibrous tumors.
7. The existence of any form of intra-uterine tumors.

With respect to sub-involution, it was very frequently met with, being a condition specially likely to occur in cases in which any form of pelvic inflammation follows delivery. It may occur also after abortion. The earliest symptom of sub-involution, and the most common is, undoubtedly, menorrhagia, a symptom nearly invariably present. Dr. Atthill, however, narrated a case of sub-involution of the uterus, in which amenorrhœa existed. The uterus in this case was very large, the sound penetrating to the depth of five inches. This patient was perfectly cured, the treatment adopted being the introduction up to the fundus of the uterus of eight grains of the solid nitrate of silver, which, dissolving, stimulated the whole of the inner surface of the uterus, and caused healthy interstitial absorption to be set up. Dr. Atthill advocated this plan of treatment in cases of enlargement of the uterus depending on sub-involution. The occurrence of enlargement of the uterus from sudden suppression of menstruation was next dwelt on; the occurrence of this form of enlargement is difficult to verify. Dr. Atthill, however, stated that he believed it to take place not unfrequently, and gave the particulars of a case in which the measurement of the uterus, by means of the sound, proved this to be so. All writers mention the liability of the uterus to enlarge during an attack of acute inflammation of that organ. The correctness of this was confirmed by the details of cases in which, by the introduction of the sound, the increase in size and subsequent diminution was proved, in instances both of metritis and peritonitis. Chronic inflammation of the uterus appears to be a very common cause of enlargement of the uterus, and gives rise to much distress. It is often associated with retroflexion, not unfrequently being the cause of that displacement. Menstruation was stated to be, in the writer's opinion, generally much diminished by the occurrence of chronic inflammation. Of all the cases of enlargement of the uterus, simple hypertrophy of the muscular tissues of the uterus is that giving rise to the greatest amount of distress, and the form least capable of being benefited by treatment; in it menstruation occasionally becomes painful, sometimes scanty, but seldom, if ever, increased in quantity. Cases illustrating this and each of the other forms of enlargement of the uterus, were detailed and commented on, and the paper concluded with some general observations on treatment. The consideration of the other forms of enlargement of the uterus were deferred to some future occasion.—*Med. and Surg. Reporter.*

### *A New Iodine Paint.*

I have been requested by some professional *confréres* to bring under the notice of the profession, a new *iodine paint*, which I have had prepared and used with satisfaction and success, in the cases of glandular enlargements and scrofulous diseases, wherein iodine is called into requisition. In the hands of esteemed and eminent practical surgeons, it has proved equally beneficial as in my own practice, and they speak or write in flattering terms of it to me.

I rub down half an ounce of iodine and a like quantity of iodide of ammonium in a Wedgwood mortar, and gradually dissolve it in twenty ounces of rectified spirit; to this I add four ounces of glycerine, shaking the solution well together. A very nice paint is thus obtained, which has the following advantages:

1. The iodine is prevented escaping, owing to the combination which, in the form of ordinary tincture, in warm weather it is very apt to do.

2. It preserves the iodide of ammonium instead of iodide of potassium; the former being a more powerful absorbent than the latter, which recent investigation has verified.

3. The action of the glycerine is soothing to the skin, keeping it soft and pliable—a contrast to the shriveling of cuticle produced by the ordinary tincture in common use, which frequently acts as a vesicant. But where absorption is desired, the part affected and its neighborhood influenced, as well as the system generally by iodine, and no local irritation required, this combination in form of paint will be found superior to the old tincture.

I have not confined the use of the preparation alone to glandular swellings or scrofulous gatherings. I have employed it in chronic cutaneous diseases, to nodes, over enlarged livers, diseased joints, to hypertrophied parts or morbid growths, and in cases wherein it was necessary to alter an abnormal action or promote absorption, and the result was uniformly satisfactory, and I think I may safely say the effect of the iodine was more really appreciable, and more quickly demonstrated in its action on the system generally, as well as by its absorbent properties locally, than the old tincture of the British Pharmacopœia, *minus* its disadvantages—J. WARING-CURRAN.—*Canada Medical Journal.*

### *On Subnitrate of Bismuth in Cholera Infantum.*

Having noticed in the November No. of the *American Practitioner* an article by Dr. Walling on the use of subnitrate of bismuth in the treatment of cholera infantum, I take pleasure in adding my testimony to the powers of this remedy in the bowel affections of children. I have not used the remedy in that stage of the disease in which Dr. Walling found it so beneficial. In the acute form of this affection, characterized by frequent vomiting and purging, I usually had but little trouble in controlling it in from six to twenty-four hours with this:

| | | | |
|---|---|---|---|
| ℞. | Plumbi. Acet., - - - - - - - | gr. x. | |
| | Morph. Acet., - - - - - - - - - | gr. ss. | |
| | Aquæ Destil., - - - - - - - | ℥ j. | |
| | Syrup. Simpl., - - - - - - - - - | ℥ ss. | M. |

Of this, I gave to children from one to two years old, a teaspoonful after each act of vomiting; or the following, which, though less generally efficient than the foregoing, I found to act promptly in some cases:

| | | |
|---|---|---|
| ℞. | Tr. Opii, - - - - - - - - - | gtt. xv. |
| | Potass. Bicarb, - - - - - - - | gr. xxiv. |
| | Syr. Simpl, - - - - - - - - - | ℥ ss. |
| | Aquæ Menth. Pip., - - - - - - - | ℥ j. M. |

Dose as the other. I sometimes gave at intervals of four hours, and repeated it a few times, half a grain of calomel, a grain of aromatic powder, and two grains of bicarbonate of soda; but I am not sure that I saw any good from it.

In cases tending to collapse, I used brandy, chloroform, quinine, coffee, and milk, with external warmth, sinapisms, etc. Where there was no disposition to collapse, the mother's milk was the only food allowed. Where the child was weaned, milk, to which lime-water was added, was given.

A certain proportion of these patients convalesced at once, after the violence of the attack was subdued, and recovery was complete in a few days; while in a large number the disease showed a marked tendency to become chronic. Add to these cases those that were not seen until after they became chronic, constituting more than half of all that I saw, and we have the class in which the subnitrate of bismuth seemed to possess such decided efficiency. In my hands it has contributed more in such cases to restore the normal condition of the mucous membrane of the alimentary tract than any other medicine that I have employed. Since I commenced its use I have relied on it mainly. In private practice and in the Western Dispensary I have seen and treated during the past season, between thirty-five and forty cases, a majority of which, as before stated, were not seen until they had become chronic. In some, the accession of the disease had been marked by acute vomiting and purging, while in others the condition had been developed gradually. Several had marked degree of emaciation and debility that seemed to render them hopeless.

Exclusive of the cases in which complete recovery followed the acute stage in from three to six days, I used the bismuth in every instance. It seemed to act beneficially alike in those that had been marked at the outset by severe vomiting and purging, in those that had been developed more gradually, in those in which the trouble seemed to be kept up by indigestion alone, and in those where inflammation of the mucous membrane existed. I gave the medicine usually as follows:

| | | |
|---|---|---|
| ℞. | Bismuth Subnit., - - - - - - - | ʒ j. |
| | Syr. Zinzib., - - - - - - - - | ʒ iij. |
| | Tr. Cinnam., - - - - - - - - | ʒ j. |
| | Tr. Opii, - - - - - - - - - - | gtt. xviij. |
| | Syr. Acaciæ, - - - - - - - - | ℥ j. M. |

Of this mixture I ordered a teaspoonful four times daily to children from one to two years old. This was continued until all disposition to vomiting had disappeared, and the fecal dejections had diminished in frequency and become more consistent, which very generally obtained in from three days to a week, sometimes sooner. After this, the medicine was given at longer intervals for a few days, and then discontinued. In some cases I added pepsin to the prescription with apparent benefit. Quinine was exhibited in those cases where a malarial element seemed to be present.

Alimentation entered as an important element into the treatment of the chronic cases, especially where emaciation and debility were marked. I did not confine these little sufferers to a milk diet; on the contrary, they were encouraged to take animal essences and soups, soft-boiled eggs, egg-nog, and even solid meats; ripe fruits and vegetables were not inhibited when the patient showed a desire for them. Alcoholic stimulants were employed in proportion to the tendency to exhaustion.

The length of time that any of these patients were under treatment was not noted. With several of them the symptoms returned, and more than once I had to resume the use of the bismuth after it had been suspended. All recovered; some in ten days to two weeks, others requiring a longer time; and with a few, owing to frequent relapses consequent on the want of proper care, complete restoration to health was delayed for two or three months.—C. K. Alexander, M.D.—*American Practitioner.*

### *Cinchona in Java.*

According to Professor Hasskarl, the cultivation of cinchona in Java continues to be a success, the weather having been favorable and the growth of the plant perfectly satisfactory. The number of plants obtained from seeds and layers was about one and a half million, principally of the species *C. calisaya;* eight hundred and seventy thousand were transplanted in addition, and over one thousand pounds of the dry bark were sent to Holland in 1869, bringing from thirty-six to fifty-four cents per pound. The total product of 1870, is estimated at eight thousand eight hundred pounds for exportation, besides some hundreds for home use in the island.—*Agricultural Report.*

